# Multi-gene panel testing increases germline predisposing mutations’ detection in a cohort of breast/ovarian cancer patients from Southern Italy

**DOI:** 10.3389/fmed.2022.894358

**Published:** 2022-08-11

**Authors:** Marcella Nunziato, Federica Di Maggio, Matilde Pensabene, Maria Valeria Esposito, Flavio Starnone, Carmine De Angelis, Alessandra Calabrese, Massimiliano D’Aiuto, Gerardo Botti, Sabino De Placido, Valeria D’Argenio, Francesco Salvatore

**Affiliations:** ^1^CEINGE–Biotecnologie Avanzate, Naples, Italy; ^2^Department of Molecular Medicine and Medical Biotechnologies, University of Naples Federico II, Naples, Italy; ^3^Department of Clinical Medicine and Surgery, University of Naples Federico II, Naples, Italy; ^4^Department of Oncology and Hematology, Regional Reference Center for Rare Tumors, Azienda Ospedaliera Universitaria (AOU) Federico II of Naples, Naples, Italy; ^5^Division of Breast Surgery, Department of Breast Disease, National Cancer Institute, Istituti di Ricovero e Cura a Carattere Scientifico (IRCCS) “Fondazione G. Pascale,” Naples, Italy; ^6^Clinica Villa Fiorita, Aversa, Italy; ^7^Division of Breast Oncology, National Cancer Institute, Istituti di Ricovero e Cura a Carattere Scientifico (IRCCS) “Fondazione G. Pascale,” Naples, Italy; ^8^Scientific Directorate, National Cancer Institute, Istituti di Ricovero e Cura a Carattere Scientifico (IRCCS) “Fondazione G. Pascale,” Naples, Italy; ^9^Department of Human Sciences and Quality of Life Promotion, San Raffaele Open University, Rome, Italy

**Keywords:** breast cancer, ovarian cancer, multigene panel, DNA repair, NGS sequencing, predictive medicine, genomic predisposition to disease

## Abstract

Breast cancer is the most common neoplasia in females worldwide, about 10% being hereditary/familial and due to DNA variants in cancer-predisposing genes, such as the highly penetrant *BRCA1/BRCA2* genes. However, their variants explain up to 25% of the suspected hereditary/familial cases. The availability of NGS methodologies has prompted research in this field. With the aim to improve the diagnostic sensitivity of molecular testing, a custom designed panel of 44 genes, including also non-coding regions and 5’ and 3’ UTR regions, was set up. Here, are reported the results obtained in a cohort of 64 patients, including also few males, from Southern Italy. All patients had a positive personal and/or familial history for breast and other cancers, but tested negative to routine *BRCA* analysis. After obtaining their written informed consent, a genomic DNA sample/patient was used to obtain an enriched DNA library, then analyzed by NGS. Sequencing data analysis allowed the identification of pathogenic variants in 12 of tested patients (19%). Interestingly, *MUTYH* was the most frequently altered gene, followed by *RNASEL, ATM, MSH6, MRE11A*, and *PALB2* genes. The reported resultsreinforce the need for enlarged molecular testing beyond *BRCA* genes, at least in patients with a personal and familial history, strongly suggestive for a hereditary/familial form. This gives also a hint to pursue more specific precision oncology therapy.

## Introduction

In 2020, breast cancer (BC) was the most common cancer in females worldwide accounting for 47.8% of total cancers and a total of 2,261,419 new cases diagnosed in the same year (24.5%). A similar trend has been observed also in Italy, where 55,133 (13.3%) new cases of BC have been registered just in 2020^1^ (last access on January 2022) (1). Fortunately, mortality has fallen sharply over a decade due to both an even more capillary diffusion of screening programs, allowing for early diagnosis, and the availability of more efficient therapeutic strategies. Male BC is rare but very aggressive, and accounts for less than 1% of all BC cases (2).

Even if the vast majority of BCs are sporadic, about 5–10% of them are considered as hereditary and due to germline predisposing variants in cancer-related genes. The highly penetrant genes associated to hereditary BC are the well-known *BRCA1* and *BRCA2* (3, 4); however, it has been shown that pathogenetic variants in these 2 genes account up to 25% of all the suspected hereditary cases, thus suggesting that other genes have to be involved in this process (5).

In the last decades, the evolution of DNA sequencing technologies, through the advent and subsequent massive diffusion of next generation sequencing (NGS)-based approaches, has enhanced the study of the molecular bases of human diseases allowing clarifying the contribution of single genes to specific diseases onset (6). In particular, the parallel testing of multiple genes, through the use of multi-gene panels, has let the simultaneous and rapid analysis of cancer predisposing genes (high-, moderate- and low-penetrance genes just known in the literature, as well as new predisposing ones) in the attempt to depict a more precise picture of the molecular basis of familial BCs and correctly identify all the at-risk subjects within the affected families (7–10).

Based on these studies, the National Comprehensive Cancer Network (NCCN) guidelines for hereditary BC introduced in 2019 the possibility to additionally test 18 different genes, beyond *BRCA1* and *BRCA2*. In particular, each of these genes has been classified as showing a very strong, strong, or just limited evidence for an increased risk of breast, ovarian, pancreatic and other cancers (11).

Moreover, recent studies have reported that a total of about 11% of all pathogenic germline variants in Caucasian patients and about 9% in Asians (affected patients) affect genes different from the traditional *BRCA1/2* (12). In particular, new evidences are emerging regarding the contribution of other genes involved in DNA damage repair mechanisms, like *PALB2, CHEK2, ATM, BRIP1*, and others (11, 12).

It is important to underline that the early identification of germline, cancer-predisposing DNA variants plays an important role, especially in breast and ovarian cancers, both for the management of the affected patients (to drive the most proper surgical and also pharmacological approaches), as well as for the implementation of prevention programs for the at-risk family members. Thus, an enlarged molecular test may be advisable in routine diagnostic settings to increase the mutations’ carriers’ identification rate (13, 14).

In this context, a custom multi-gene panel, including 44 genes already known in the recent literature as related prevalently to breast, ovarian, colon and prostate cancers predisposition, was set up. With the aim to improve the diagnostic sensitivity of hereditary BCs molecular screening, a cohort of 64 *BRCA1/2*-negative, breast and ovarian cancer patients were analyzed through the above-mentioned multi-genes, custom panel enrichment method followed by NGS. Pathogenic variants in not routinely tested genes were identified. The results reported herein highlight once again the need for more enlarged molecular testing to improve the management of BC patients and of their families.

## Materials and methods

### Patients’ enrollment

A total of sixty-four individuals, including 57 cancer patients (54 women and 3 men), and seven unaffected women with a positive family history for breast and/or ovarian cancer, were totally enrolled in the present study among those attending to CEINGE Biotecnologie Avanzate laboratories (Napoli, Italy) to carry out the germline *BRCA1/2* molecular screening from 2014 to 2018. Before the diagnostic procedure, a genetic counseling was offered to the patients in the reference clinical centers, the senology units of both “Istituto Nazionale dei Tumori–Fondazione G. Pascale” and of the Federico II University (Napoli, Italy), to assess their personal and familial cancer history and verify the presence of the criteria to be admitted to molecular testing, according to national and international guidelines (Italian Association of Medical Oncology, AIOM, NCCN). At this time, clinical data, including personal and family history, and the pedigree of each patient were collected.

**FIGURE 1:**
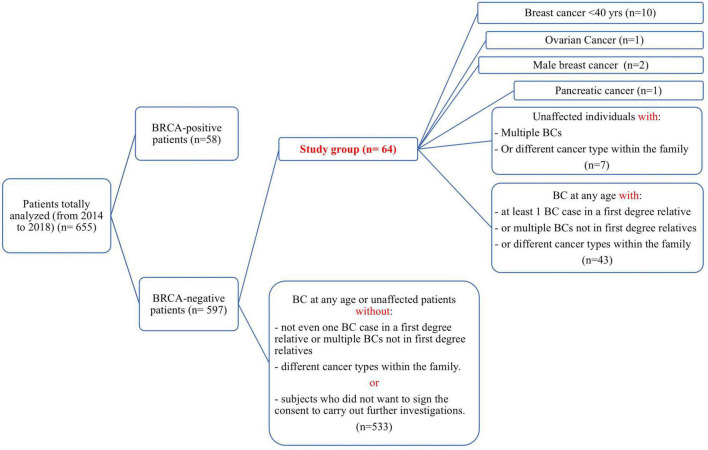
Strobe diagram representing the study design and the inclusion criteria over an observation time of 5 years. For each category, the corresponding number of subjects is reported in parentheses.

The patients selected for the present study were all negative for the detection of single nucleotide substitutions and small insertion/deletions (INDELs) in *BRCA1* and *BRCA2* genes. In the course of the study, the *BRCA1/2* molecular testing was integrated with the analysis also of the CNVs (Copy Number Variants) through the MLPA (Multiplex ligation Probe amplification) method, that was available for almost all patients, 54/64 selected for the present study. During the 5 years of observation time, a total of 655 subjects, 58 being carriers of a *BRCA1/2* pathogenic variant, was analyzed. Among the remaining 597 tested negative, the patients with at least one of the following inclusion criteria were selected: (i) early onset BC (under-forty years); (ii) BC at any age but with at least one BC case in a first degree relative, or multiple BCs not in first degree relatives, or different cancer types within the family; (iii) ovarian cancer; (iv) male BC; (v) pancreatic cancer; (vi) unaffected individuals with multiple BCs or different cancer types within the family (Figure 1). All patients come from the Campania region in Southern Italy. Demographic features of the obtained study groups are summarized in Table 1, while the characteristics of each study participant are detailed in the Supplementary Table 1. Unfortunately, in this cohort of patients in our area, we have not had the possibility to analyze the relatives for being mutation carriers because of reluctance to undergo the test, since now.

**TABLE 1:** Demographic summary of the study group.*

	Number (*n*)	Percentage (%)
**Gender**		
Female	61	95.3
Male	3	4.7
**Cancer**		
Yes	57	89
No	7	11
**Cancer type**		
Female breast cancer	53	82.8
Ovarian cancer	1	1.6
Male breast cancer	2	3.1
Pancreatic cancer	1	1.6
Unaffected	7	10.9
**Age of onset**		
<40 years	12	18.8
>40 years	45	70.3
Not applicable	7	10.9

*Detailed data of each study participant are reported in Supplementary Table 1.

All patients gave their written informed consent to the study that was carried out according to the tenets of the Helsinki Declaration and approved by the Istituto Nazionale Tumori–Fondazione G. Pascale Ethics Committee (protocol number 3 of 03/25/2009).

Genomic DNA (gDNA) was extracted from peripheral blood samples using the Maxwell 16 Blood LEV DNA Purification Kit (#AS1290–Promega Corporation, WI, United States), according to manufacturer’s instructions. DNA quantity was evaluated using the QuBit 3.0 fluorimeter (#Q32850–Thermo Fisher Scientific, MA, United States), while DNA quality was assessed by 0.8% agarose gel electrophoresis.

### Multi-gene panel custom design

A custom panel was chosen as methodological approach and designed with the aim to possibly highlight novel candidate genes not yet associated to hereditary BC risk. Thus, genes not included in the currently commercially available kits and for which no guidelines regarding the clinical management of the carriers have been released, were selected and analyzed in the present study. For the same reason, during the patients’ selection step the co-occurrence of different cancers within the families was carefully evaluated.

Based on these criteria, the genes to be included in the custom NGS panel were carefully selected after a deep study of the literature (9, 15, 16). Next, the custom design was realized using the web-based application HaloPlex SureDesign, we previously used for the study of other genetically heterogeneous diseases (17). The resulting custom multi-genes panel accounts for a total of 44 target genes, being equivalent to 804 target regions and 16,978 probes for a total target size of 416,043 kbp. The panel includes all the coding exons of each gene and 50 bp at exon boundaries on each side (5’ and 3’). Specifically, it comprehends genes chosen after a careful study of recent literature, in detail: 26 genes are mainly related to breast/ovarian cancers; 14 genes are related to colon cancer and four genes are related to prostate cancer (all these neoplasia are present and often mixed in the same family in the Campania population). The list of the genes included in the final panel is provided in Table 2.

**TABLE 2:** List of the genes included in the custom panel.

Genes included in the panel (*n* = 44)				
**N°**	**Genes**	**Name**	**ClinGen database gene-disease validity**	**RefSeq**
1	*AKT1*	v-akt murine thymoma viral oncogene homolog 1	None	NM_005163 NP_005154
2	*ATM*	ATM serine/threonine kinase	Familial ovarian cancer, hereditary non-polyposis colon cancer, hereditary breast carcinoma, ataxia telangiectasia	NM_000051 NP_000042
3	*AXIN2*	Axin 2	Oligodontia-cancer predisposition syndrome	NM_004655 NP_004646
4	*BARD1*	BRCA1 associated RING domain 1	Hereditary breast carcinoma, hereditary non-polyposis colon cancer, familial ovarian cancer	NM_000465 NP_000456
5	*BMPR1A*	Bone morphogenetic protein receptor type IA	Generalized juvenile polyposis/juvenile polyposis coli	NM_004329 NP_004320
6	*BRCA1*	Breast cancer 1, Early onset	Breast-ovarian cancer, familial, susceptibility to, Fanconi anemia	NM_007294 NP_009225
7	*BRCA2*	Breast cancer 2, Early onset	Breast-ovarian cancer, familial, susceptibility to, Fanconi anemia	NM_000059 NP_000050
8	*BRIP1*	BRCA1 interacting protein C-terminal helicase 1	Hereditary breast carcinoma, familial ovarian cancer, Fanconi anemia	NM_032043 NP_114432
9	*CDH1*	Cadherin 1, type 1	Hereditary non-polyposis colon cancer, hereditary breast carcinoma, familial ovarian cancer, hereditary diffuse gastric adenocarcinoma	NM_004360 NP_004351
10	*CDKN2A*	Cyclin-dependent kinase inhibitor 2A	Melanoma-pancreatic cancer syndrome	NM_000077 NP_000068
11	*CHEK2*	Checkpoint kinase 2	Hereditary breast carcinoma, familial ovarian cancer, Fanconi anemia, hereditary non-polyposis colon cancer	NM_001005735 NP_001005735
12	*ELAC2*	elaC ribonuclease Z 2	None	NM_018127 NM_173717 NP_060597 NP_776065
13	*EPCAM*	Epithelial cell adhesion molecule	Hereditary breast carcinoma, colorectal cancer, hereditary non-polyposis	NM_002354 NP_002345
14	*FAM175A*	Family with sequence similarity 175 member A	None	NM_139076 NP_620775
15	*FANCC*	Fanconi anemia, complementation group C	Fanconi anemia	NM_000136 NP_000127
16	*GREM1*	Gremlin 1, DAN family BMP antagonist	Hereditary mixed polyposis syndrome	NM_001191323 NP_001178252
17	*HOXB13*	Homeobox B13	None	NM_006361 NP_006352
18	*KLLN*	killin, p53-regulated DNA replication inhibitor	None	NM_001126049 NP_001119521
19	*MLH1*	MutL Homolog 1	Hereditary breast carcinoma, colorectal cancer, hereditary non-polyposis, mismatch repair cancer syndrome	NM_000249 NP_000240
20	*MLH3*	mutL homolog 3	Hereditary non-polyposis colon cancer	NM_001040108 NP_001035197
21	*MRE11A*	MRE11 homolog A, double strand break repair nuclease	Hereditary breast carcinoma, familial ovarian cancer	NM_005591 NP_005582
22	*MSH2*	MutS Homolog 2	Hereditary breast carcinoma, Lynch syndrome, mismatch repair cancer syndrome	NM_000251 NP_000242
23	*MSH6*	MutS Homolog 6	Hereditary breast carcinoma, Lynch syndrome, mismatch repair cancer syndrome	NM_000179 NP_000170
24	*MSR1*	Macrophage scavenger receptor 1	None	NM_138716 NP_619730
25	*MUTYH*	MutY DNA glycosylase	MUTYH-related attenuated familial adenomatous polyposis, hereditary breast carcinoma, familial ovarian cancer	NM_001048172 NP_001041637
26	*NBN*	Nibrin	Hereditary breast carcinoma, Nijmegen breakage syndrome	NM_002485 NP_002476
27	*NF1*	Neurofibromin 1	Familial ovarian cancer, neurofibromatosis	NM_001042492 NP_001035957
28	*PALB2*	Partner and localizer of BRCA2	Hereditary non-polyposis colon cancer, hereditary breast carcinoma, familial ovarian cancer, Fanconi anemia	NM_024675 NP_078951
29	*PIK3CA*	Phosphatidylinositol-4,5-bisphosphate 3-kinase, catalytic subunit alpha	Hereditary breast carcinoma, familial ovarian cancer, overgrowth syndrome and/or cerebral malformations due to abnormalities in MTOR pathway genes	NM_006218 NP_006209
30	*PMS2*	PMS1 homolog 2, mismatch repair system component	Hereditary breast carcinoma, Lynch syndrome, mismatch repair cancer syndrome	NM_000535 NP_000526
31	*POLD1*	Polymerase (DNA directed), delta 1, catalytic subunit	Polymerase proofreading-related adenomatous polyposis	NM_001256849 NM_002691 NP_001243778 NP_002682
32	*POLE*	Polymerase (DNA directed), epsilon, catalytic subunit	Polymerase proofreading-related adenomatous polyposis	NM_006231 NP_006222
33	*PTEN*	Phosphatase and tensin homolog	PTEN hamartoma tumor syndrome	NM_000314 NP_000305
34	*RAD50*	RAD50 homolog, double strand break repair protein	Hereditary breast carcinoma, familial ovarian cancer	NM_005732 NP_005723
35	*RAD51C*	RAD51 paralog C	Fanconi anemia, hereditary breast carcinoma, familial ovarian cancer	NM_058216 NP_478123
36	*RAD51D*	RAD51 paralog D	Hereditary breast carcinoma, familial ovarian cancer	NM_002878 NP_002869
37	*RINT1*	RAD50 Interactor 1	Hereditary breast carcinoma, familial ovarian cancer	NM_021930 NP_068749
38	*RNASEL*	Ribonuclease L	None	NM_021133 NP_066956
39	*SCG5*	Secretogranin V	None	NM_001144757 NP_001138229
40	*SMAD4*	SMAD family member 4	Generalized juvenile polyposis/juvenile polyposis coli	NM_005359 NP_005350
41	*SMARCA4*	SWI/SNF related, matrix associated, actin dependent regulator of chromatin, subfamily a, member 4	Hereditary non-polyposis colon cancer, Coffin-Siris syndrome, rhabdoid tumor predisposition syndrome	NM_003072 NP_003063
42	*STK11*	serine/threonine kinase 11	Familial ovarian cancer, Peutz-Jeghers syndrome	NM_000455 NP_000446
43	*TP53*	Tumor Protein P53	Familial ovarian cancer, Li-Fraumeni syndrome 1	NM_000546 NM_001126112 NP_000537 NP_001119584
44	*XRCC2*	X-ray repair complementing defective repair in Chinese hamster cells 2	Hereditary breast carcinoma, familial ovarian cancer	NM_005431 NP_005422

### Libraries preparation and sequencing

DNA libraries were obtained using the HaloPlex Target Enrichment System (#G9911C Agilent Technologies, CA, United States), following manufacturer’s instructions. The first step of the used capture methodology is a genomic DNA digestion, carried out using 16 different restriction enzymes provided by the company. Next, after the hybridization step, thousands of different targets are amplified in the same reaction using the custom specific probes, and each sample is uniquely indexed, allowing for pooling and sequencing several samples all together. More in detail, firstly, the quantity of each gDNA was assessed using the QuBit dsDNA BR Assay kit, and 225 ng of each gDNA were fragmented using eight different restriction reactions incubated 30 min at 37°C. The eight digestion reactions were, then, combined into a single hybridization mix tube (one tube/patient) that contains target-specific probes and the unique sample index.

The hybridization reaction was performed incubating the samples at 95°C for 10 min, and then at 54°C for 16 h. Next, the hybridized DNA fragments, containing biotin, were captured using streptavidin beads and a DNA ligase was added to the capture reaction to close the gaps in the circularized target DNA-probe hybrids. Finally, a PCR mix for the captured target DNA amplification step was prepared, followed by the elution with NaOH and a subsequent magnetic beads purification, using AMPure XP beads (#A63880–Beckman Coulter, Fullerton, CA, United States). The profile of each enriched library was assessed using the TapeStation 2200 using the High Sensitivity D1000 assay (#5067-5584/85), and the library quantity was measured using QuBit 3.0. Libraries were first diluted each at 10 nM and then pooled together to ensure equimolarity between samples. The final concentration of the libraries pool was 4 nM in a single tube ready to be sequenced.

Eight different sequencing runs were performed using the Illumina MiSeq platform (#MS102-2002–PE 150 × 2, San Diego, CA, United States). Eight pM of the denatured final libraries pool were combined to 25% of 8 pM PhiX, and were loaded into the MiSeq reagent cartridge, according to manufacturer’s instructions.

### Bioinformatic analysis and Sanger confirmation

The sequencing platform used in this study produces raw data in FASTQ files format ready for download. FASTQ files are univocally assigned to each patient thanks to the index sequences added to gDNAs during the libraries’ preparation procedure. Data analysis was performed using the Agilent’s SureCall v4.2.0, a bioinformatic free tool that align and call all the variants found. All potentially clinical-relevant variants were confirmed using Sanger Sequencing using the same sample where NGS was performed. The method described herein is able to detect single nucleotide variants and small INDELs. This statement is based on: (i) the comparison of the variants found in *BRCA1/2* by a CE_IVD *BRCA1/2* test (10) and the proposed method, which showed a 100% concordance; and (ii) the confirmation by Sanger sequencing of all the potentially clinically-relevant variants identified in this study, which confirmed all of them. Since the largest INDELs identified in this study group and confirmed at Sanger comprises five nucleotides, the data presented in this paper allow to assess this as maximum number, even if we cannot exclude that it may correctly detect larger INDELs (18). Indeed, previous studies comparing different algorithms to assess INDELS-calling from NGS data report a good positive predictive value for INDELs less or equal to 10 nucleotides.

For all the variants identified, the allele frequency was verified using the gnomAD database (v.2.1.1, accessed on May 2022), the frequencies are reported in Tables 3, 4. Furthermore, different prediction tools were set up to assess pathogenicity of the variants found: namely, ClinVar (accessed on January 2022), VarSome v11.1 of December 21st 2021 (19), Alamut^®^ software suite, Mutation Taster, ACMG (American College of Medical Genetics)/AMP (Association for Molecular Pathology) criteria and, where applicable, Human Splicing Finder v.3.1. The Deleterious Annotation of genetic variants using Neural Networks (DANN) and the Combined Annotation Dependent Depletion (CADD) scores were assessed for each variant found (Tables 3, 4); finally, BayesDel and Rare Exome Variant Ensemble Learner (REVEL) tools were used to evaluate all the variants of uncertain significance (VUSs) found (Table 4).

**TABLE 3:** Pathogenic variants found in genes different from the BRCAs.

Patient^#^	Gene	cDNA^†^	Protein^†^	Reference SNP ID	Variation type	ClinVar database	ACMG/AMP^§^ criteria	Allele frequency (gnomAD)	CADD score*	ClinGen**
P12 (Figure 3)	*ATM*	c.1463G > A	p.(Trp488Ter)	rs879254093	Non-sense	Pathogenic	Pathogenic	0.0000319	38	**Definitive:** hereditary breast carcinoma, ataxia telangiectasia; **Moderate:** hereditary non-polyposis colon cancer; **Limited:** familial ovarian cancer
P14 (Figure 7)	*MSH6*	c.892C > T	p.(Arg298Ter)	rs146816935	Non-sense	Pathogenic	Pathogenic	0.00000796	35	**Definitive:** mismatch repair cancer syndrome, Lynch syndrome; **Disputed:** hereditary breast carcinoma
P15 (Figure 6D)	*MUTYH*	c.849 + 3A > C	p.(?)	rs587780751	Splicing Site	Conflicting interpretations of pathogenicity P (15); VUS (1)	Likely pathogenic	0.0000743	23.2	**Definitive:** MUTYH-related attenuated familial adenomatous polyposis; **Moderate**: MUTYH-related attenuated familial adenomatous polyposis; **Limited:** familial ovarian cancer; **No Known Disease Relationship:** hereditary breast carcinoma
P21 (Figure 6A), P39 (Figure 6B)	*MUTYH*	c.1103G > A	p.(Gly368Asp)	rs36053993	Missense	Pathogenic	Pathogenic	This variant does not have a gnomAD entry	32	
P24 (Figure 6C), P52 (Figure 6E)	*MUTYH*	c.452A > G	p.(Tyr151Cys)	rs34612342	Missense	Pathogenic	Pathogenic	0.00154	26.8	
P59 (Figure 5)	*PALB2*	c.1727_1731 delGTAAT	p.(Ser576fs)	nr	Frameshift	Not Reported	Likely pathogenic	This variant does not have a gnomAD entry	–	**Definitive:** Fanconi anemia complementation group N, hereditary breast carcinoma; **Moderate:** familial ovarian cancer; **Limited:** hereditary non-polyposis colon cancer
P13 (Figure 4A), P54 (Figure 4B), P32 (Figure 4C)	*RNASEL*	c.793G > T	p.(Glu265Ter)	rs74315364	Non-sense	Conflicting interpretations of pathogenicity LB (1); VUS (1)	Pathogenic	0.00356	34	No data available

^#^One patient carrying the variant c.1100T > G in *MRE11A* gene, is not reported in this table, since the variant is classified as VUS in ClinVar database, but as likely pathogenic according to ACMG/AMP criteria.

^†^Variants’ nomenclature according to Human Genome Variation Society (HGVS) guidelines.

^§^ACMG, American College of Medical Genetics, and AMP, Association for Molecular Pathology; nr, not reported; P, pathogenic; LB, likely benign; VUS, uncertain significance variant.

*CADD score, Combined Annotation Dependent Depletion (CADD) tool scores may predict how is deleterious a single nucleotide variant by the integration of multiple annotations. Scores above 30 are considered ‘likely deleterious’ and scores below 30 may be considered ‘likely benign’.

**ClinGen: https://search.clinicalgenome.org.

**FIGURE 2:**
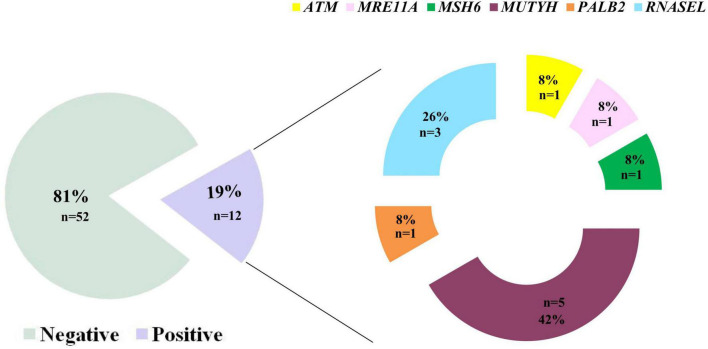
Results of the multi-gene panel testing based on NGS-approach in this study group (*N* = 63 totally analyzed patients). Eighty-three% of patients were found not to be carriers of pathogenic variants, while 17% was positive for the presence of a clinically interesting mutation. Interestingly, we highlight that MUTYH carried most of the variants identified herein (46%) followed by the RNASEL gene (27%).

**FIGURE 3:**
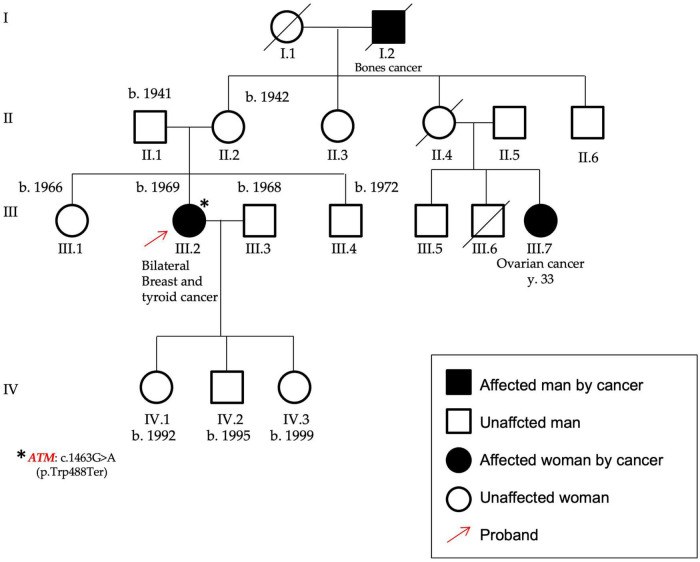
Pedigree of P12 carrying the variant c.1463G > A, p.(Trp488Ter), found in the ATM gene. The patient was affected by bilateral breast cancer and thyroid cancer and her maternal cousin by early onset ovarian cancer. The variant identified in this proband (highlighted by the red arrow) was a nonsense mutation in the ATM gene, as identified by the asterisk.

**FIGURE 4:**
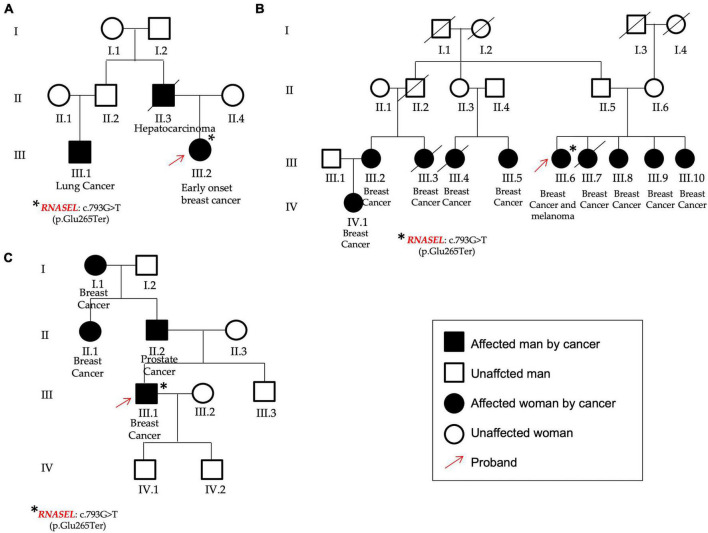
Pedigrees of P13 **(A)**, P54 **(B)**, and P32 **(C)** patients found to carry the same variant in the *RNASEL* gene. **(A)** The patient was affected by early onset breast cancer and showed different cases of hepatocarcinoma and lung cancer; **(B)** The patient affected by breast cancer and melanoma showed other cases of breast cancer from both the paternal and the maternal branches. **(C)** The male patient was affected by breast cancer and declared other cases both for breast and prostate cancers. Red arrows identified the three probands, asterisks the *RNASEL* gene variant.

**FIGURE 5:**
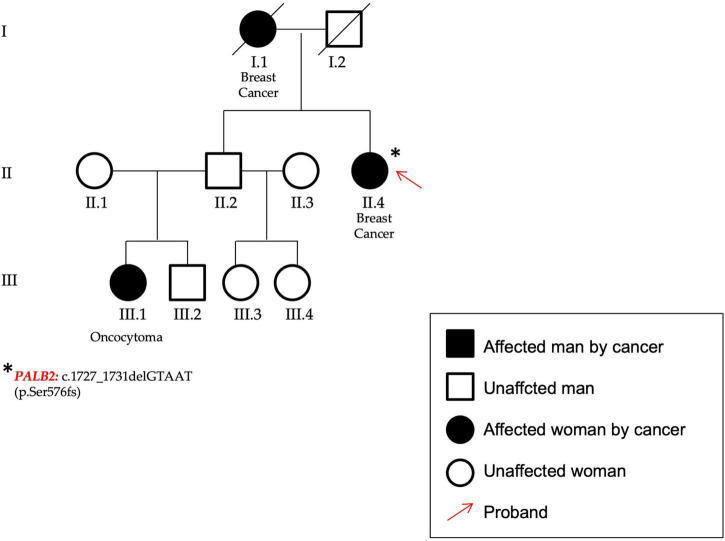
In figure the pedigree of P59. The patient (identified by a red arrow) affected by breast cancer showed a frameshift variant in PALB2 gene.

**FIGURE 6:**
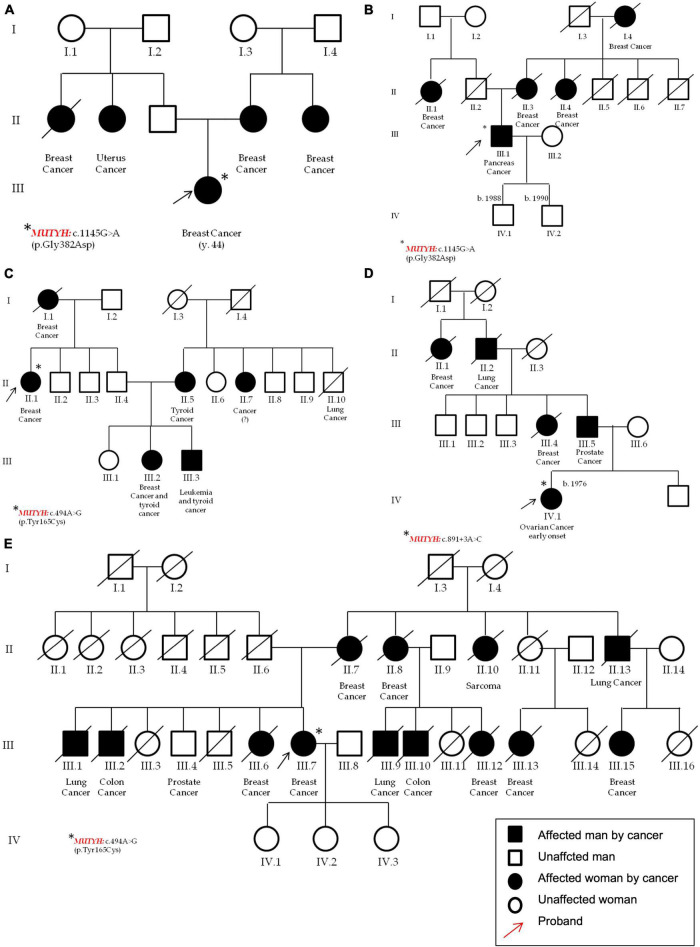
Pedigrees of the patients that carry pathogenic variants in the MUTYH gene. In detail, in **(A)** (P21) and **(B)** (P39) one female and one male that carry the c.1103G > A p.(Gly368Asp); in **(C)** (P24) a patient with c.452A > G, p.(Tyr151Cys); and in **(D)** (P52) a female with the c.891 + 3A > C. Pedigree **(E)** (P15) of the second patient carrying the MUTYH variant c.452A > G, p.(Tyr151Cys). All the probands are highlighted by red arrows and the asterisks identifies MUTYH different variants.

**FIGURE 7:**
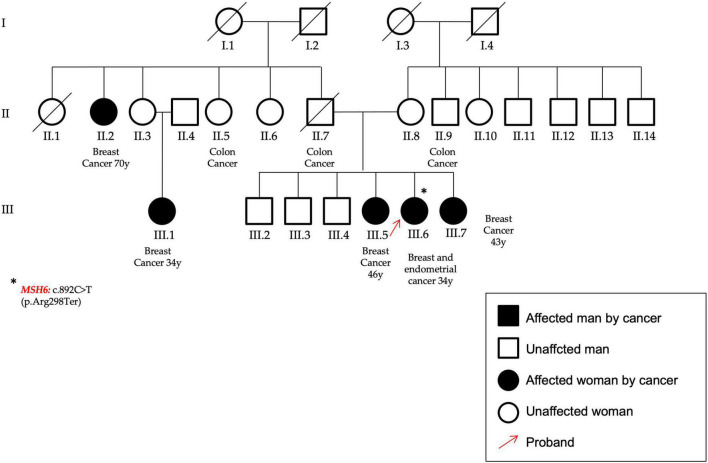
In the figure the pedigree of the patient (P14) that carry the variant identified in the MSH6 gene, c.892C > T–p.(Arg298Ter). She had an early onset BC and a subsequent endometrial cancer; moreover, other cases of both breast and colon cancers are reported within her family. The proband was highlighted by a red arrow and the asterisk identifies the MSH6 gene mutation.

**TABLE 4:** List of the 33 variants of unknown significance (VUSs) identified in 23 patients (36%).

N°	Gene acronym	cDNA^†^	Protein^†^	ClinVar database	Reference SNP ID	Disease-related (MedGen, Orphanet, OMIM)	ACMG/AMP^§^ criteria	DANN^#^ score	Allele frequency (gnomAD)	CADD* score	BayesDel** prediction	REVEL*** prediction
1	*MRE11A*	c.1958C > G	p.(Ser653Trp)	Uncertain significance	rs587782166	Hereditary cancer-predisposing syndrome	Uncertain Significance	0.93	0.000003978	36	Tolerated	Benign
2	*STK11*	c.*413C > A	p.(?)	Uncertain significance	rs533940465	Peutz-Jeghers syndrome	Benign	0.88	0.00003210	15.09	No data available	No data available
3	*MRE11A*	c.1100T > G	p.(Val367Gly)	Uncertain significance	rs749526614	Hereditary cancer-predisposing syndrome	Likely pathogenic	0.9969	0.00001594	32	Damaging	Pathogenic
4	*ATM*	c.3857G > T	p.(Cys1286Phe)	Uncertain significance	rs876660770	Hereditary cancer-predisposing syndrome	Uncertain significance	0.9918	0.000003980	25.4	Damaging	Pathogenic
5	*AXIN2*	c.1633G > A	p.(Gly545Arg)	Uncertain significance	rs148951121	Oligodontia-colorectal cancer syndrome	Uncertain significance	0.9429	0.00003538	20.2	Tolerated	Benign
6	*MSH6*	c.3383A > T	p.(Tyr1128Phe)	Uncertain significance	rs587779261	Hereditary cancer-predisposing syndrome	Uncertain Significance	0.9173	0.00001193	14.13	Tolerated	No data available
7	*RAD50*	c.2092A > G	p.(Ile698Val)	Conflicting interpretations of pathogenicity LB (1), VUS (1)	rs781213977	Hereditary cancer-predisposing syndrome	Uncertain Significance	0.9957	0.000003979	26.1	Tolerated	Benign
8	*TP53*	c.*772delT	p.(?)	Uncertain significance	rs200757381	Li-Fraumeni syndrome	Benign	–	This variant does not have a gnomAD entry	–	No data available	No data available
9	*RAD50*	c.3857T > C	p.(Phe1286Ser)	Uncertain significance	rs587781369	–	Uncertain significance/LP	0.9925	This variant does not have a gnomAD entry	31	Damaging	Pathogenic
10	*MSH6*	c.892C > G	p.(Arg298Gly)	Uncertain significance	rs146816935	Lynch syndrome Hereditary cancer-predisposing syndrome Hereditary non-polyposis colon cancer	Uncertain significance	0.9853	0.000003980	35	Tolerated	Benign
11	*RINT1*	c.388G > A	p.(Ala130Thr)	Uncertain significance	rs138345617	–	Uncertain significance	0.8851	0.0003535	7.5	Tolerated	Benign
12	*MRE11A*	c.*656_*659 dupTCTC	p.(?)	Uncertain significance	rs201800515	Ataxia-telangiectasia-like disorder 1	Benign	–	This variant does not have a gnomAD entry	–	No data available	No data available
13	*RAD50*	c.1744A > G	p.(Ser582Gly)	Uncertain significance	rs747324016	Hereditary cancer-predisposing syndrome	Uncertain significance	0.9907	0.00001194	23	Tolerated	Benign
14	*MSH2*	c.128A > G	p.(Tyr43Cys)	Conflicting interpretations of pathogenicity LB (4), VUS (9)	rs17217723	Lynch syndrome Hereditary cancer-predisposing syndrome Hereditary non-polyposis colon cancer	Uncertain significance/LP	0.9972	0.00007272	32	Damaging	Pathogenic
15	*ATM*	c.1444A > C	p.(Lys482Gln)	Conflicting interpretations of pathogenicity LB (3), VUS (8)	rs202173660	Ataxia-telangiectasia syndrome Hereditary cancer-predisposing syndrome	Uncertain significance	0.9877	0.00008841	36	Tolerated	Benign
16	*NBN*	c.1570G > A	p.(Glu524Lys)	Uncertain significance	rs774324419	Hereditary cancer-predisposing syndrome	Uncertain significance	0.9111	0.000007973	33	Tolerated	Benign
17	*NF1*	c.*3248G > A	p.(?)	Uncertain significance	rs527971565	Neurofibromatosis, familial spinal Café-au-lait macules with pulmonary stenosis	Uncertain significance	0.9173	This variant does not have a gnomAD entry	14.9	No data available	No data available
18	*MSH6*	c.4002-10T > A	p.(?)	Conflicting interpretations of pathogenicity B (2); LB (3), VUS (6)	rs545466048	Lynch syndrome Hereditary non-polyposis colon cancer	Uncertain significance	0.8064	0.0002572	8	No data available	No data available
19	*MUTYH*	c.319G > A	p.(Ala107Thr)	Uncertain significance	rs369854269	MYH-associated polyposis Hereditary cancer-predisposing syndrome	Uncertain significance	0.9992	0.00003581	27.3	Damaging	Pathogenic
20	*MRE11A*	c.1643T > C	p.(Ile548Thr)	Conflicting interpretations of pathogenicity LB (1), VUS (4)	rs373522639	Hereditary cancer-predisposing syndrome	Uncertain significance/B	0.8851	0.00008846	20.6	Tolerated	Benign
21	*MSH6*	c.3079G > A	p.(Gly1027Ser)	Uncertain significance	rs587779264	Lynch syndrome Hereditary cancer-predisposing syndrome	Uncertain significance/LP	0.9985	This variant does not have a gnomAD entry	31	Damaging	Pathogenic
22	*MSH6*	c.361C > A	p.(Leu121Ile)	Uncertain significance	rs587781657	Lynch syndrome Hereditary cancer-predisposing syndrome	Uncertain significance	0.9574	0.000007073	23.3	Tolerated	Benign
23	*RAD50*	c.3836G > A	p.(Arg1279His)	Uncertain significance	rs375710541	Hereditary cancer-predisposing syndrome	Uncertain significance/LP	0.9993	0.00004599	29.7	Damaging	Benign
24	*POLD1*	c.2052G > C	p.(Gln684His)	Conflicting interpretations of pathogenicity LB (4), VUS (6)	rs144143245	Colorectal cancer 10 Hereditary cancer-predisposing syndrome	Benign	0.9972	0.0004372	25	Tolerated	Benign
25	*BRIP1*	c.2087C > T	p.(Pro696Leu)	Uncertain significance	rs147755155	Familial cancer of breast Fanconi anemia, complementation group J Hereditary cancer-predisposing syndrome	Uncertain significance/LP	0.9608	0.00005659	29.7	Damaging	Pathogenic
26	*CHEK2*	c.862C > T	p.(Pro288Ser)	Conflicting interpretations of pathogenicity LB (1), VUS (9)	rs587780179	Familial cancer of breast Hereditary cancer-predisposing syndrome	Uncertain significance	0.4956	0.00009477	10.6	Tolerated	Benign
27	*MRE11A*	c.1784-4C > T	p.(?)	Uncertain significance	rs768257868	Hereditary cancer-predisposing syndrome	Uncertain significance	0.4303	0.00001197	3.9	No data available	No data available
28	*KLLN*	c.-906C > G	p.(?)	Uncertain significance	rs587782079	Hereditary cancer-predisposing syndrome	Uncertain significance	0.6567	0.00003192	9	No data available	No data available
29	*PTEN*	c.*1583G > A	p.(?)	Uncertain significance	rs548599209	PTEN hamartoma tumor syndrome	Benign	0.8143	0.0006757	6.6	No data available	No data available
30	*RAD50*	c.1153C > T	p.(Arg385Cys)	Uncertain significance	rs139372231	Hereditary cancer-predisposing syndrome	Uncertain significance	0.9984	0.00001769	25.4	Tolerated	Benign
31	*RINT1*	c.1940C > T	p.(Ser647Leu)	Uncertain significance	rs187666745	–	Uncertain significance	0.9989	0.00006364	39	Damaging	No data available
32	*SMAD4*	c.*5578G > C	p.(?)	Uncertain significance	rs867684157	Myhre syndrome Osler hemorrhagic telangiectasia syndrome Juvenile Polyposis	Benign	0.3417	0.004694	3.2	No data available	No data available
33	*NF1*	c.7055A > G	p.(Asn2352Ser)	Uncertain Significance	rs763082717	Neurofibromatosis, type 1 Hereditary cancer-predisposing syndrome	Likely Benign	0.9958	0.00001989	25.2	Tolerated	Benign

^†^Variants’ nomenclature according to Human Genome Variation Society (HGVS) guidelines.

^§^ACMG, American College of Medical Genetics, and AMP, Association for Molecular Pathology.

^#^DANN, deleterious annotation of genetic variants using neural networks. Last accession on databases January 2022.

*CADD score, Combined Annotation Dependent Depletion (CADD) tool (see also the legend of Table 3).

**BayesDel is a deleteriousness meta-score, here we reported the prediction based on tool’s scores (ranges from –1.29334 to 0.75731).

***REVEL is a tool that combines scores from 13 different individual tools for predicting the pathogenicity of variants, here we reported the prediction based on the tool’s scores.

## Results

All the 64 patients selected for this study were analyzed as described under Methods section. An average of 3,643,168 reads/sample with a mean reads’ depth in the target regions of 878X, was obtained. Sequencing data/sample are reported in the Supplementary Table 2.

The results reported herein showed that 28 patients (44%) were negative for multi-gene panel testing, 12 patients presented pathogenic/likely pathogenic variants (19%), and 23 patients (36%) showed potentially interesting variants currently classified as VUSs in ClinVar database.

Interestingly, this extended molecular screening allowed the identification of pathogenic variants in 12 different, unrelated patients (19%) resulted negative at *BRCA1/2* test (concordance of mutations found in previous *BRCA1/2* test was of 100%). These variants span over 6 different genes: *ATM, RNASEL, PALB2, MSH6, MRE11A*, and *MUTYH* genes, *MUTYH* carrying most of them (three different variants in five patients) (Figure 2 and Table 3).

In particular, as reported in Table 3, a total of eight pathogenic variants was found, of which three were nonsense variants, three were missense, one was an intronic variant affecting a splicing site, and one was a frameshift variant in the *PALB2* gene, not reported in the ClinVar database, but classified as pathogenic according to the ACMG/AMP criteria (20).

In the *ATM* gene (NM_000051), a nonsense variant [c.1463G > A, p.(Trp488Ter)–rs879254093] in the exon 10 was found in a female patient affected by bilateral BC and also by a thyroid cancer (P12). She showed also a case of ovarian cancer (maternal cousin) (Figure 3). This variant is predicted to cause loss of normal protein function through the protein truncation or nonsense-mediated mRNA decay; loss-of-function variants in ATM gene are known in the literature to be pathogenic (21, 22).

The *RNASEL* (NM_021133.3) nonsense variant c.793G > T, p.(Glu265Ter) (rs74315364) in the exon 2, was found in three unrelated patients: two females (P13 and P54) and one male (P32).

This gene has been already associated to hereditary prostate cancer and it has been reported that men carrying this variant show a median age at prostate cancer onset 11 years lower respect to the not-carriers (23). In this study group, one of the two females that presented the *RNASEL* gene variant was affected by early onset BC, showed a case of hepatocarcinoma (her father) and lung cancer too (her paternal cousin) among her family members (P13, Figure 4A). The other female was affected by BC and melanoma and presented nine other cases of breast cancer in the family (four sisters, four cousins, and one nephew) (P54, Figure 4B). Finally, the same variant was identified in a man (P32) affected by BC and showing other cases of oncological diseases not only for BC (mother and grandmother), but also for prostate cancer (his father) (Figure 4C).

In the *PALB2* (NM_024675.3) gene a frameshift variant not previously reported in any database was found. The variant c.1727_1731delGTAAT p.(Ser576Lysfs*8) was classified according to ACMG/AMP criteria (20) as certainly pathogenic. The patient was affected by bilateral BC and showed other cases of breast and renal cancers within the family (P59, Figure 5).

*MUTYH* (NM_001048174.2) harbors most of the variants identified in this cohort. Three different variants were found in five unrelated patients; moreover, the c.1103G > A p.(Gly368Asp)—rs36053993—and the c.452A > G, p.(Tyr151Cys)—rs34612342—variants were found twice, both being very common not only in familial adenomatous polyposis, but also in the general population. In particular, the *MUTYH* p.(Gly368Asp) and p.(Tyr151Cys) variants are well-established pathogenic variants for *MUTYH*-related polyposis and are estimated to account for 50–82% of *MUTYH*-associated polyposis in European patients (24). In this study, the p.(Gly368Asp) was found in a female patient affected by BC and with other cases of breast and uterus cancers in her family (P21, Figure 6A), and in a man affected by pancreatic cancer with other cases of BC in his pedigree (P39, Figure 6B).

The second *MUTYH* variant is the c.452A > G, p.(Tyr151Cys) (rs34612342) and was identified in two women. One was affected by bilateral BC and showed different cases of breast, thyroid, and leukemia (P24, Figure 6C); the second patient was affected by BC and declared other cases of breast, lung, prostate and bones cancers (P52, Figure 6E), respectively. The third *MUTYH* variant was the c.849 + 3A > C (rs587780751). This variant affects a non-conserved intronic nucleotide. Mutation Taster predicts a damaging outcome for this variant, and Alamut algorithms predict the variant to alter normal splicing. In this study, the c.849 + 3A > C (rs587780751) in the *MUTYH* gene was found in a patient with early onset ovarian cancer and with other cases of breast, prostate and lung cancers (P15, Figure 6D).

A nonsense nucleotide substitution in the *MSH6* gene (NM_000179.3) was found in a patient affected by BC (onset at 34 years of age), who developed an endometrial cancer at follow-up. She showed other cases of BC and also for colon cancer (P14, Figure 7).

Finally, one variant identified in the *MRE11A* gene (NM_005591.3) an interesting VUS based on ACMG/AMP criteria (20). The *MRE11A* variant c.1100T > G–p.(Val367Gly) (rs749526614) is currently reported as variant of uncertain significance in ClinVar database (three entries, last accession on January 2022). Indeed, it affects a highly conserved amino acidic residue but the impact of this glycine to valine substitution on protein functions is not yet established since no functional studies have been published so far to assess its consequences. On the other side, the variant has a low allelic frequency and several prediction tools suggest a deleterious effect, DANN score being 0.99, and a score of 32 obtained by using the Combined Annotation Dependent Depletion (CADD score), deleterious and pathogenic also for BayesDel and REVEL tool’s predictions, respectively (Table 4).

In addition to the above-mentioned pathogenic variants a total of 33 rare VUSs identified using ClinVar database were additionally found (see also the allele frequency obtained using gnomAD database in Table 4); 7 (about 22%) were predicted to be benign/likely benign; and the remaining 26 (about 78%) were considered VUSs, according to the ACMG/AMP criteria (20).

## Discussion

Breast cancer is still the most frequent cancer in females worldwide and an important cause of cancer death (1). Early diagnosis represents the major factor able to impact BC outcome and the overall patients’ survival (25). In this context, the identification of the hereditary forms, related to germline, inherited DNA variants, is crucial to admit patients and their at-risk family members to the most proper surveillance and therapeutic programs. In particular, the availability of specific drugs effective in mutated patients has increased the request for extensive molecular testing in the presence of breast/ovarian cancers diagnosis (26). Accordingly, thanks to the diffusion of NGS-based techniques in routine diagnostic laboratories, today the molecular analysis of the *BRCA1/2* genes, including single nucleotide substitutions, small INDELs, and also CNVs evaluation can be performed in a few days (27, 28). Nevertheless, though the well-established role of the *BRCA1/2* genes, both of them explain up to 25% of all the suspected hereditary forms (14). So far, several studies have been carried out to fill-in this gap and identify other predisposing genes by investigating different size of genes panels (9, 29–34). Even if *BRCA1/2* remain the highly penetrant genes, some evidences are accumulating, especially regarding other genes involved in DNA repair mechanisms, so that, a couple of years ago, the NCCN guidelines for hereditary cancers have been updated to include their analysis (35). Taking into consideration cohorts of patients closer to the one present in this work, positive rates of about 15% were found (about 10% in *BRCA1/2* and about 5% in other genes) (31–33). Nevertheless, more recent studies show a higher fraction of non-*BRCA1/2* genes mutations (11, 14) principally due to the inclusion of lower-risk patients and larger multi-gene panels tested. Based on all these data and on the observation of the frequent co-occurrence of different cancer types (not only breast and ovarian, but also colon, melanoma, pancreas and prostate cancers) within the affected families, a custom panel including 44 genes to be simultaneously analyzed through an enrichment-based protocol followed by NGS analysis was set up. Here, are reported the results obtained by the implementation of this panel to analyze a cohort of 64 subjects from Southern Italy. All these subjects were already admitted to *BRCA1/2* molecular testing based on their personal and/or familial history, but tested negative. As a consequence, they were the ideal candidates for an extended molecular analysis. Interestingly, 12 additional individuals that carry pathogenic/likely pathogenic variants, being equivalent to 19% of the analyzed subjects, were identified. This result highlights the importance of enlarged molecular testing to correctly identify hereditary BCs due to DNA variants in moderate or low penetrance genes. Indeed, without this analysis, these subjects would have been considered not mutated and their cancer risk would be considered like that of the general population. In this way, instead, their inherited cancer-predisposition was identified, allowing for their better clinical management and offering the opportunity to identify the other at-risk subjects within their families.

In particular, pathogenic/likely pathogenic variants in six genes were found according to ClinVar database and ACMG/AMP classification using VarSome tool: three of which being in well-established genes associated to breast and/or ovarian cancer (*ATM, PALB2* and *MSH6*) and 3 (*MRE11A, MUTYH* and *RNASEL*) being in good candidacy to became susceptibility genes (36).

*ATM* (ataxia-telangiectasia mutate) encodes a protein kinase involved in the cellular response to DNA double-strand breaks through the phosphorylation of several proteins, including *BRCA1* (37). *ATM* gene homozygous or compound heterozygous DNA variants are associated to the onset of an autosomal recessive disease, namely ataxia telangiectasia, featured by a progressive cerebellar degeneration and oculo-cutaneous telangiectasia (37). Interestingly, *ATM* heterozygous variants have been associated to an increased risk of BC so that *ATM* is considered a moderate penetrance gene for hereditary breast and ovarian cancers (37–39). An *ATM* truncating variant was found in a patient with bilateral BC and a previous thyroid cancer; moreover, other cancer cases are reported within her family.

*PALB2* (partner and localizer of BRCA2) encodes a protein crucial for the correct functions of both BRCA1 and BRCA2 (39, 40). While *PALB2* bi-allelic variants have been associated to Fanconi’s anemia, monoallelic loss-of-function variants have been reported to be responsible for an increased risk of both breast and pancreatic cancers (41). Moreover, even if *PALB2* mutation carriers’ frequency is low and varies across populations (between 1 and 2.5%), their overall BC risk has been reported to be similar to that for *BRCA2* mutation carriers (42). A *PALB2* gene variant, the c.1727_1731delGTAAT p.(Ser576LysfsTer8) was found in a patient affected by BC with other cases of different oncological diseases. This variant has not been previously reported; however, it has been classified as pathogenic according to ACMG/AMP guidelines and different prediction tools. Even if functional studies are required to establish its pathogenicity, since it is a deletion of five nucleotides determining at protein level a premature stop codon, its pathogenicity is plausible.

*MSH6* (Mut S Homolog 6) encodes a member of the mismatch repair genes family whose pathogenic variants have been associated to Lynch syndrome (43). It has been recently assessed that *MSH6* variants are associated also to an increased risk of BC, suggesting their testing in the at-risk families (39, 44). The nonsense variant was found in a female affected by BC and endometrial cancer at the age of 34 years; with three cases of colon cancer and five cases of BC in her family.

*MUTYH* was the most frequently mutated gene in this population, since three different pathogenic variants in five unrelated patients (two variants were found twice, see Results section for more details) were detected. Mammalian MutY homologue (*MUTYH*) encodes a DNA glycosylase involved in base excision repair during DNA replication and DNA damage repair (44). Inherited bi-allelic variants in this gene have been associated to the *MUTYH*-associated polyposis (MAP), which is a risk factor for colorectal cancer development (45). In particular, the p.(Gly368Asp) and the p.(Tyr151Cys) variants, found also in this study, are the most common in the Caucasian population, accounting for about 75% of the pathogenic variants detected in the adenomatous polyposis patients (46). A large meta-analysis defined that *MUTYH* bi-allelic variants are associated to a 28-fold increased risk for colorectal cancer, while monoallelic variants had a limited effect (47). This may be due to several factors, including other co-occurring risk factors, such as the age of onset (48). In addition, due to its crucial role in DNA errors correction, a *MUTYH* involvement in predisposing also other cancers has been proposed (48).

Currently, the role of *MUTYH* monoallelic variants in BC patients is quite controversial with some studies showing an association and others demonstrating that no link exists (49–54). More recently, the use of enlarged genomic testing in cancer patients has shown that *MUTYH* monoallelic variants are identified in *BRCA1/2*-negative BC patients, even if with a frequency similar to that of the expected carrier frequency (55–57). *MUTYH* mono-allelic variants may act as low-penetrant BC predisposing factors, thus implying that additional concomitant risk factors, like age, ethnicity, or environmental factors, are required to determine a clinical phenotype. Based on all the above, NCCN guidelines for “Genetic/Familial High-Risk Assessment: Breast, Ovarian, and Pancreatic” (version 2.2022, accessed on March 2022), includes *MUTYH* mono-allelic variants within the lower penetrance genes that may be added as part of multi-gene testing, but for which the association with BC is still insufficient. However, increasing knowledge about the frequency of *MUTYH* variants and their spectrum of associated cancers may provide in the future an additional instrument for patients’ risk stratification and for the development of novel therapies (58, 59). It has to be mentioned that Rizzolo et al. (60), carrying out a multicenter study on male BC risk in Italy, found that *MUTYH* pathogenic variants were associated to an increased cancer risk (60). Moreover, a recent study by Doddato et al. (36), by analyzing an Italian cohort of 200 individuals, identified *MUTYH* pathogenetic variants in 4 unrelated subjects (36). Even if further studies on larger groups of patients are required to confirm these data, these studies, together with the results obtained herein, suggest that *MUTYH* mono-allelic variants may be a currently under-estimated risk-factor for BC cancer predisposition, at least in the Southern Italian population.

The most frequent variant identified in this study group was the nonsense variant c.793G > T, p.(Glu265Ter) in the *RNASEL* gene found in three unrelated subjects. *RNASEL* (endoribonuclease or RNase L) encodes an enzyme involved in antiviral and anti-proliferate pathways (61). Germline variants in this gene have been associated to an increased risk of prostate cancer and it has been suggested as susceptibility gene also for other cancers (61–63). In particular, the *RNASEL* p.(Glu265Ter) variant was firstly described in prostate cancer patients and, since a reduction of protein activity was measured, a protein loss of function was hypothesized as pathogenetic mechanism (64). Thus, even if the role of this variant is still unclear, it was proposed as a rare founder allele in the Caucasian population (62). In the study group presented herein, three unrelated patients were found to carry the *RNASEL* p.(Glu265Ter) variant. No one carried other pathogenic variants in other genes and, interestingly, one of them was a male BC case whose father was affected by prostate cancer. As discussed above, the clinical significance of this variant is still controversial; moreover, as for the variants identified in *MUTYH*, the p.(Glu265Ter) is frequent in the Caucasian population. This finding is intriguing but, on the basis of current knowledge, it is not possible to define whether this is a casual association or whether there is a causal link.

In addition to the pathogenic variants, as usual in the case of extended molecular analysis, also several VUSs were identified using ClinVar database. One of these variants may be classified as pathogenic according to the ACMG/AMP guidelines (20), CADD score, BayesDel and REVEL predictions; it is a missense nucleotide change in the *MRE11A* gene involved in the repair of double-strand breaks and thus associated to breast and ovarian cancers (65). Other seven variants seem to be benign/likely benign, while the remaining 26 were classified as VUSs also in ACMG/AMP guidelines. Furthermore, the calculation of the CADD score to obtain *in silico* predictions, showed that nine variants could be considered harmful, but only four of these have pathogenicity predictions also for the BayesDel and REVEL tools, although are still VUSs for the ACMG/AMP criteria (66). On the one hand, these findings are currently difficult to interpret and may give inconclusive and frustrating results for patients, nevertheless their detection and annotation is crucial to increase the scientific knowledge and improve their correct classification, on the other hand those are frustrating also for patients’ relatives especially in case of family history of cancers. They are usually not tested for, but they know the existence and the possible consequences of being a mutation carrier.

Several studies have been carried out so far analyzing variable in size panels of genes and patients’ cohort (9, 29, 30). All together these studies are allowing to evaluate the prevalence of predisposing DNA variants in non-*BRCA1/2* genes and to highlight some specific genes and/or variants with a higher frequency in specific geographic areas or population. Moreover, since a lot of variants with scarcely known clinical significance are emerging, accumulating genomic data is required to further classify them.

The study reported herein aims to contribute to this field by adding the data obtained by the analysis of patients from Southern Italy. In the present study, 5/64 analyzed patients carried a *MUTYH* monoallelic variant; this rate is so high and so unusual to be worth reporting and to admit the possibility that *MUTYH* variants may have a higher penetrance in the South-Italian genetic background or lifestyle/environment (67). In conclusion, the presented data support the use of enlarged multigene testing to increase the detection rate of hereditary BCs and to highlight also less frequently mutated genes and/or recurring variants in specific populations. Moreover, additional genetic variants acting as risk modifiers may be also highlighted allowing for a better BC patients stratification based on their genotype. The precision therapy goal is more and more important in these days to reach advancement also in progressively severe clinical situations.

## Study limitations

A limitation of the study is that it was not able to measure weak (or even moderate) associations between relatively common variants (in *MUTYH* gene) and a relatively common disease (female BC) due to the small cohort. Therefore, larger studies followed by statistical data analysis are required to confirm this hypothesis. Similar issues apply also to the RNASEL p.(Glu265Ter) variant. In the present study, this variant was found in three unrelated patients allowing to speculate an intriguing association. Further studies are required to assess this hypothesis and exclude that repeated observations, of the same variant, may be due simply to random chance. Moreover, it is also possible that other variants not detectable by the used strategy (deeper intronic regions, CNVs in the non-*BRCA1/2* genes, or other genes not included in the panel) may be associated to the patients’ phenotype.

## Data availability statement

The original contributions presented in this study are publicly available at: https://www.ncbi.nlm.nih.gov/bioproject/PRJNA820693/. This data can be found here: NCBI Bioproject, accession no. PRJNA820693.

## Ethics statement

The studies involving human participants were reviewed and approved by Istituto Nazionale Tumori–Fondazione G. Pascale Ethics Committee (protocol number 3 of 03/25/2009). The patients/participants provided their written informed consent to participate in this study.

## Author contributions

FSa and MN: conceptualization and overall supervision of methodology, data collection and discussion, project planning, and data analysis. MN, FD, VD’A, MVE, and FSt: methodology and experimental procedure and analysis of results. MP, CD, AC, MD’A, GB, and SD: patients’ enrollment, clinical data collection, and histopathology analysis. FSa and VD’A: writing and final editing of the manuscript. All authors contributed for manuscript writing also by drafting some relevant parts including tables and figures and read and agreed to the published version of the manuscript.

## Footnotes

1
https://gco.iarc.fr/today/
