# Supplementary material for: Multi-gene panel testing increases germline predisposing mutations’ detection in a cohort of breast/ovarian cancer patients from Southern Italy
Source: Front Med (Lausanne). 2022 Aug 11;9:894358. doi: 10.3389/fmed.2022.894358 (PMC9403188; doi:10.3389/fmed.2022.894358)
Supplement: Supplementary file 1 [file Table_1.DOCX]

Supplementary Material

**Supplementary Table 1.** Clinical features of the 64 patients enrolled in the study.

| ***Patient ID*** | ***Sex*** | | ***Personal cancer history*** | | | | | | ***Familial cancers*** | | ***BRCA CNVs analysis*** | | ***Pathogenic/Likely Pathogenic variants identified*** | | | ***Menarche Age*** | ***Pregnancy*** | | ***Contraceptives*** | | ***Smoking*** | ***Obesity*** | |  |
| --- | --- | --- | --- | --- | --- | --- | --- | --- | --- | --- | --- | --- | --- | --- | --- | --- | --- | --- | --- | --- | --- | --- | --- | --- |
|  |  |  | ***Cancer type*** | | ***Age of onset*** | | ***Additional cancer(s) onset*** | |  |  |  |  |  |  |  |  |  |  |  |  |  |  |  |  |
| P1 | F | | Breast | | 43 | | - | | Breast | | No | |  | | | 12y | 2 | | No | | No | No | |  |
| P2 | F | | - | | NA | | - | | Ovarian | | Yes | |  | | | 12y | 1 abortion | | No | | Yes | No | |  |
| P3 | F | | Breast | | 65 | | - | | Breast | | No | |  | | | - | No | | - | | No | No | |  |
| P4 | F | | - | | NA | | - | | Breast | | Yes | |  | | | 11y | No | | - | | No | No | |  |
| P5 | F | | Breast | | 63 | | - | | Melanoma, Breast | | No | |  | | | - | 2 | | - | | No | No | |  |
| P6 | F | | Breast | | 49 | | - | | Pancreas, Breast | | Yes | |  | | | 12y | 1 | | Yes | | No | No | |  |
| P7 | F | | - | | NA | | - | | Prostate, Breast | | Yes | |  | | | 12y | 2 | | Yes | | Yes | No | |  |
| P8 | F | | Breast | | 44 | | - | | Breast, Uterous | | Yes | |  | | | 13y | 3 | | No | | No | No | |  |
| P9 | F | | Breast | | 39 | | - | | Breast | | Yes | |  | | | 13y | 2 | | No | | No | No | |  |
| P10 | F | | Breast | | 35 | | - | | Liver, Breast | | Yes | |  | | | 9y | 1 | | n.d | | n.d | n.d | |  |
| P11 | F | | Breast | | 52 | | - | | Breast, Colon, Brain | | Yes | |  | | | 10y | 1 | | Yes | | No | No | |  |
| P12 | F | | Breast | | 46 | | Tyroid, Bilateral Breast | | Ovarian, Bones | | Yes | | *ATM* (NM_000051), c.1463G>A, p.(Trp488Ter), rs879254093 | | | 13y | 3 | | No | | No | Yes | |  |
|  |  | |  | | | | | |  | |  | |  | | |  |  | |  | |  |  | |  |
|  |  | |  | | | | | |  | |  | |  | | |  |  | |  | |  |  | |  |
| ***Patient ID*** | ***Sex*** | | ***Personal cancer history*** | | | | | | ***Familial cancers*** | | ***BRCA CNVs analysis*** | | ***Pathogenic/Likely Pathogenic variants identified*** | | | ***Menarche Age*** | ***Pregnancy*** | | ***Contraceptives*** | | ***Smoking*** | ***Obesity*** | |  |
|  |  |  | ***Cancer type*** | | ***Age of onset*** | | ***Additional cancer(s) onset*** | |  |  |  |  |  |  |  |  |  |  |  |  |  |  |  |  |
| P13 | F | | Breast | | 38 | | - | | Lung, Hepatocarcinoma | | Yes | | RNASEL (NM_021133.3), c.793G>T, p.(Glu265Ter), rs74315364 | | | 14y | No | | No | | No | No | |  |
| P14 | F | | Breast | | 49 | | Endometrial Carcinoma | | Breast, Colon | | Yes | | MSH6 (NM_000179.3), c.892C>T, p.(Arg298Ter), rs146816935 | | | 12y | No | | Yes | | Yes | No | |  |
| P15 | F | | Ovarian | | 39 | | - | | Prostate,Breast, Lung | | Yes | | MUTYH (NM_001048174.2), c.849+3A>C, (rs587780751) | | | 11y | No | | Yes | | No | No | |  |
| P16 | F | | Breast | | 39 | | Breast Cancer relapse | | Breast Cancer, Larynx cancer, Leukemia, Prostate cancer | | Yes | |  | | | 14y | 2 | | No | | No | No | |  |
| P17 | F | | Breast | | 53 | | - | | Breast, Stomach, Pancreas, Liver | | Yes | |  | | | 10y | 1 | | Yes | | Yes | No | |  |
| P18 | F | | Breast | | 56 | | - | | Pancreas, Brain, Breast, Lung | | Yes | |  | | | 11y | 2 | | No | | No | No | |  |
| P19 | F | | Breast | | 68 | | - | | Breast, Prostate, testicular carcinoma, ovarian | | Yes | |  | | | 11y | 3 | | No | | No | No | |  |
| P20 | F | | - | | NA | | - | | Colon, breast, lung | | Yes | |  | | | 12y | No | | No | | No | n.d | |  |
| ***Patient ID*** | ***Sex*** | | ***Personal cancer history*** | | | | | | ***Familial cancers*** | | ***BRCA CNVs analysis*** | | ***Pathogenic/Likely Pathogenic variants identified*** | | | ***Menarche Age*** | ***Pregnancy*** | | ***Contraceptives*** | | ***Smoking*** | ***Obesity*** | |  |
|  |  |  | ***Cancer type*** | | ***Age of onset*** | | ***Additional cancer(s) onset*** | |  |  |  |  |  |  |  |  |  |  |  |  |  |  |  |  |
| P21 | F | | Breast | | 43 | | - | | Breast, Uterous | | Yes | | MUTYH (NM_001048174.2), c.1103G>A p.(Gly368Asp), rs36053993 | | | 9y | 1 | | No | | No | No | |  |
| P22 | F | | - | | NA | | - | | Breast, ovarian | | Yes | |  | | | 11y | 2 | | No | | No | No | |  |
| P23 | F | | Breast | | 68 | | - | | Breast, Prostate, Brain, Lung, Bladder | | Yes | |  | | | 12y | 4 | | No | | No | No | |  |
| P24 | F | | Breast | | 55 | | - | | Breast, tyroid and leukemia | | Yes | | MUTYH (NM_001048174.2), c.452A>G, p.(Tyr151Cys), rs34612342 | | | 13y | 2 | | No | | Yes | No | |  |
| P25 | F | | Breast | | 58 | | - | | Breast, Uterous, kidney, bowel, lung | | Yes | |  | | | 12y | 2 | | No | | No | No | |  |
| P26 | F | | Breast | | 53 | | - | | Breast, Bladder, Lung, kidney, bones | | Yes | |  | | | 13y | 2 + 1 abortion | | No | | No | No | |  |
| P27 | F | | Breast | | 51 | | - | | Linfoma, Breast, Prostate, Brain | | Yes | |  | | | n.d. | n.d. | | n.d. | | n.d. | n.d. | |  |
| P28 | F | | Breast | | 48 | | - | | Linfoma, Breast, Prostate, Brain | | Yes | |  | | | n.d. | n.d. | | n.d. | | n.d. | n.d. | |  |
| P29 | F | | Breast | | 38 | | - | | Linfoma, Breast, Prostate, Brain | | Yes | |  | | | n.d. | n.d. | | n.d. | | n.d. | n.d. | |  |
| ***Patient ID*** | ***Sex*** | | ***Personal cancer history*** | | | | | | ***Familial cancers*** | | ***BRCA CNVs analysis*** | | ***Pathogenic/Likely Pathogenic variants identified*** | | | ***Menarche Age*** | ***Pregnancy*** | | ***Contraceptives*** | | ***Smoking*** | ***Obesity*** | |  |
|  |  |  | ***Cancer type*** | | ***Age of onset*** | | ***Additional cancer(s) onset*** | |  |  |  |  |  |  |  |  |  |  |  |  |  |  |  |  |
| P30 | F | | - | | NA | | - | | Breast, Melanoma | | Yes | |  | | | 10y | 2 | | No | | Yes | No | |  |
| P31 | F | | Breast | | 39 | | - | | Breast, ovarian | | Yes | |  | | | 9y | 2 | | Yes | | No | Yes | |  |
| P32 | **M** | | Breast | | 45 | | - | | Breast, Prostate | | Yes | | RNASEL (NM_021133.3), c.793G>T, p.(Glu265Ter), rs74315364 | | | - | - | | - | | No | Yes | |  |
| P33 | F | | Breast | | 51 | | - | | Ovarian, Breast, Prostate, Larynx, Lung | | Yes | |  | | | 13y | 4 | | No | | No | No | |  |
| P34 | F | | Breast | | 62 | | - | | Breast, colon, ovarian | | No | |  | | |  |  | |  | |  |  | |  |
| P35 | F | | Breast | | 35 | | - | | Breast, Melanoma, Prostate, Lung | | Yes | |  | | | 14y | 1 extrauterine | | No | | No | No | |  |
| P36 | F | | Breast | | 45 | | - | | Breast, Bladder, lung, leukemia | | Yes | |  | | | 13y | 2+2 abortions | | No | | No | No | |  |
| P37 | F | | Breast (Triple Negative) | | 38 | | - | | Breast | | Yes | |  | | | 11y | 3 | | Yes | | No | No | |  |
| P38 | F | | Breast | | 58 | | - | | Breast, Stomach, Uterous, Lung, Bowel, Bones, Trachea | | Yes | |  | | | 12y | 2+2 abortions | | Yes | | Yes | No | |  |
| P39 | **M** | | Pancreatic | | 62 | | - | | Breast | | Yes | | MUTYH (NM_001048174.2), c.1103G>A p.(Gly368Asp), rs36053993 | | | - | - | | - | | No | No | |  |
| ***Patient ID*** | ***Sex*** | | ***Personal cancer history*** | | | | | | ***Familial cancers*** | | ***BRCA CNVs analysis*** | | ***Pathogenic/Likely Pathogenic variants identified*** | | | ***Menarche Age*** | ***Pregnancy*** | | ***Contraceptives*** | | ***Smoking*** | ***Obesity*** | |  |
|  |  |  | ***Cancer type*** | | ***Age of onset*** | | ***Additional cancer(s) onset*** | |  |  |  |  |  |  |  |  |  |  |  |  |  |  |  |  |
| P40 | **M** | | Breast | | 39 | | - | | Breast, Prostate | | Yes | |  | | | - | - | | - | | No | No | |  |
| P41 | F | | Breast | | 62 | | - | | Ovarian, Lung, Pancreas, Breast, Liver, Kidney | | Yes | |  | | | 11y | 3 | | No | | No | No | |  |
| P42 | F | | Breast | | 43 | | - | | Breast, Kidney, lung, melanoma | | Yes | |  | | | 13y | 1+3 abortions | | No | | No | No | |  |
| P43 | F | | Breast | | 55 | | - | | Breast | | Yes | |  | | | 12y | 3+1 abortion | | Yes | | Yes | No | |  |
| P44 | F | | Breast | | 58 | | - | | Breast, Liver, Colon, Bowel, Sarcoma | | Yes | |  | | | 13y | 2 | | No | | Yes | No | |  |
| P45 | F | | Breast | | 45 | | Ovarian Cancer | | Breast | | Yes | |  | | | 14y | 2+5 abortions | | No | | No | No | |  |
| P46 | F | | Breast | | 50 | | - | | Breast | | Yes | |  | | | 11y | 1 | | Yes | | No | No | |  |
| P47 | F | | Breast | | 57 | | - | | Breast | | Yes | |  | | | 11y | 3 | | No | | Yes | No | |  |
| P48 | F | | Breast | | 64 | | - | | Breast | | No | |  | | | 12y | 2 | | No | | No | No | |  |
| P49 | F | | Breast | | 53 | | Uterous Cancer | | Breast, Prostate, Brain, Liver | | Yes | |  | | | 15y | 2 | | No | | No | No | |  |
| P50 | F | | Breast | | 47 | | - | | Stomach, Breast, Prostate, Lung | | Yes | |  | | | 11y | 2 | | Yes | | Yes | No | |  |
| P51 | F | | Breast | | 65 | | - | | Breast | | Yes | |  | | | 13y | 2 | | No | | Yes | No | |  |
| P52 | F | | Breast | | 73 | | - | | Breast, Colon, Lung, Prostate and Sarcoma | | Yes | | MUTYH (NM_001048174.2), c.452A>G, p.(Tyr151Cys), rs34612342 | | | 12y | 3 | | No | | No | No | |  |
| ***Patient ID*** | ***Sex*** | | ***Personal cancer history*** | | | | | | ***Familial cancers*** | | ***BRCA CNVs analysis*** | | ***Pathogenic/Likely Pathogenic variants identified*** | | | ***Menarche Age*** | ***Pregnancy*** | | ***Contraceptives*** | | ***Smoking*** | ***Obesity*** | |  |
|  |  |  | ***Cancer type*** | | ***Age of onset*** | | ***Additional cancer(s) onset*** | |  |  |  |  |  |  |  |  |  |  |  |  |  |  |  |  |
| P53 | F | | Breast | | 36 | | - | | Breast, Colon | | Yes | |  | | | 8y | 1+1 abortion | | No | | No | No | |  |
| P54 | F | | Breast | | 53 | | Melanoma | | Breast | | Yes | | RNASEL (NM_021133.3), c.793G>T, p.(Glu265Ter), rs74315364 | | | 11y | 2+1 abortion | | No | | No | No | |  |
| P55 | F | | Breast | | 46 | | - | | Breast | | Yes | |  | | | 11y | 2 | | Yes | | Yes | Yes | |  |
| P56 | F | | - | | NA | | - | | Prostate, uterous, breast | | Yes | |  | | | 11y | 2 | | No | | Yes | No | |  |
| P57 | F | | Breast | | 44 | | - | | Breast | | No | |  | | | 15y | No | | No | | No | No | |  |
| P58 | F | | Breast | | 65 | | - | | Breast, Leukemia, Lung | | No | |  | | | 11y | 1 | | No | | No | No | |  |
| P59 | F | | Breast | | 42 | | Bilateral Breast | | Kidney, Breast | | No | | PALB2 (NM_024675.3), c.1727_1731delGTAAT p.(Ser576Lysfs*8) | | | 14y | 3+1 abortion | | No | | No | No | |  |
| P60 | F | | Breast | | 49 | | - | | Breast, uterous | | No | |  | | | 12y | 2 | | No | | No | No | |  |
| P61 | F | | Breast | | 51 | | - | | Breast, Colon, Uterous | | No | |  | | | 13y | 3+2 abortions | | No | | No | No | |  |
| P62 | F | | Breast | | 58 | | - | | Mieloma, Pancreas, Breast | | Yes | |  | | | 12y | 1 | | No | | No | No | |  |
| P63 | F | | Breast | | 32 | | - | | Breast, Bones | | Yes | |  | | | 13y | No 1 abortion | | Yes | | No | No | |  |
| P64 | F | | Breast | | 51 | | - | | Breast | | Yes | |  | | | 12y | 3 | | No | | No | No | |  |
|  | |  | |  | |  | |  | |  | |  | |  |  | | |  |  |  | | |  | |
| F: female; M: male; NA: not applicable; y: year. | | | | | | | |  | |  | |  | |  |  | | |  |  |  | | |  | |

**Supplementary Table 2**. Raw sequencing data obtained for each study subjects.

| **Patients' ID** | **Number of reads** | **Average read depth in analyzable target regions** | **Percentage of analyzable target regions covered by at least 10X** |
| --- | --- | --- | --- |
| P1 | 1.931.189 | 444 | 97,83 |
| P2 | 1.028.482 | 234 | 96,53 |
| P3 | 1.779.822 | 414 | 98,16 |
| P4 | 1.255.262 | 285 | 97,94 |
| P5 | 1.242.306 | 283 | 97,94 |
| P6 | 1.318.734 | 304 | 98,37 |
| P7 | 1.291.608 | 299 | 97,72 |
| P8 | 1.033.788 | 236 | 96,1 |
| P9 | 1.445.276 | 333 | 98,16 |
| P10 | 1.390.030 | 318 | 97,72 |
| P11 | 2.924.854 | 709 | 98,05 |
| P12 | 1.424.588 | 328 | 97,83 |
| P13 | 2.584.609 | 627 | 98,37 |
| P14 | 1.507.803 | 350 | 97,61 |
| P15 | 3.129.836 | 766 | 98,37 |
| P16 | 3.348.696 | 813 | 98,37 |
| P17 | 3.100.606 | 758 | 97,83 |
| P18 | 1.636.572 | 389 | 97,51 |
| P19 | 3.233.010 | 821 | 98,59 |
| P20 | 3.014.250 | 719 | 98,37 |
| P21 | 2.377.236 | 586 | 98,59 |
| P22 | 868.175 | 211 | 96,1 |
| P23 | 2.794.363 | 692 | 97,51 |
| P24 | 1.614.823 | 390 | 98,05 |
| P25 | 4.604.122 | 1.106 | 98,26 |
| P26 | 4.646.859 | 1.144 | 98,48 |
| P27 | 961.030 | 228 | 97,07 |
| P28 | 1.330.767 | 338 | 97,4 |
| P29 | 5.350.580 | 1.360 | 98,48 |
| P30 | 4.333.619 | 1.075 | 98,37 |
| P31 | 8.467.059 | 2.052 | 98,81 |
| P32 | 6.797.901 | 1.636 | 98,48 |
| P33 | 6.742.556 | 1.607 | 98,7 |
| P34 | 6.011.093 | 1.514 | 98,7 |
| P35 | 8.452.269 | 2.047 | 98,59 |
| P36 | 4.998.292 | 1.276 | 98,59 |
| P37 | 9.277.635 | 2.255 | 98,7 |
| **Patients' ID** | **Number of reads** | **Average read depth in analyzable target regions** | **Percentage of analyzable target regions covered by at least 10X** |
| P38 | 4.476.761 | 1.122 | 98,48 |
| P39 | 1.352.687 | 320 | 97,83 |
| P40 | 6.560.616 | 1.570 | 98,81 |
| P41 | 1.289.347 | 312 | 97,94 |
| P42 | 5.329.105 | 1.283 | 98,81 |
| P43 | 4.158.858 | 978 | 98,59 |
| P44 | 3.648.565 | 856 | 98,59 |
| P45 | 8.227.541 | 1.933 | 98,59 |
| P46 | 3.658.074 | 863 | 98,26 |
| P47 | 7.524.974 | 1.805 | 98,48 |
| P48 | 4.809.216 | 1.144 | 98,48 |
| P49 | 8.067.716 | 1.893 | 98,16 |
| P50 | 3.898.820 | 943 | 98,81 |
| P51 | 4.376.967 | 1.066 | 98,59 |
| P52 | 4.893.290 | 1.178 | 97,83 |
| P53 | 5.095.613 | 1.232 | 98,7 |
| P54 | 2.725.684 | 656 | 98,37 |
| P55 | 5.641.488 | 1.373 | 97,72 |
| P56 | 1.420.625 | 344 | 97,94 |
| P57 | 6.919.298 | 1.620 | 97,83 |
| P58 | 5.050.797 | 1.278 | 96,85 |
| P59 | 1.752.683 | 422 | 98,05 |
| P60 | 1.770.277 | 428 | 97,61 |
| P61 | 2.010.619 | 486 | 97,94 |
| P62 | 1.311.433 | 319 | 97,29 |
| P63 | 4.474.858 | 1.110 | 97,94 |
| P64 | 3.467.130 | 772 | 98,31 |
